# Explaining why childhood abuse is a risk factor for poorer clinical course in bipolar disorder: a path analysis of 923 people with bipolar I disorder

**DOI:** 10.1017/S0033291719002411

**Published:** 2020-10

**Authors:** Steven Marwaha, Paul M. Briley, Amy Perry, Phillip Rankin, Arianna DiFlorio, Nick Craddock, Ian Jones, Matthew Broome, Katherine Gordon-Smith, Lisa Jones

**Affiliations:** 1 Institute for Mental Health, University of Birmingham, Edgbaston, Birmingham, B15 2TT, UK; 2 Institute of Mental Health, University of Nottingham, Nottingham, UK; 3 Psychological Medicine, University of Worcester, Worcester, UK; 4 Brighton and Sussex University Hospitals Trust, Brighton, UK; 5 National Centre for Mental Health, Cardiff University, Cardiff, UK

**Keywords:** Affective instability, bipolar disorder, childhood abuse, impulsivity, path analysis

## Abstract

**Background:**

Childhood abuse is a risk factor for poorer illness course in bipolar disorder, but the reasons why are unclear. Trait-like features such as affective instability and impulsivity could be part of the explanation. We aimed to examine whether childhood abuse was associated with clinical features of bipolar disorder, and whether associations were mediated by affective instability or impulsivity.

**Methods:**

We analysed data from 923 people with bipolar I disorder recruited by the Bipolar Disorder Research Network. Adjusted associations between childhood abuse, affective instability and impulsivity and eight clinical variables were analysed. A path analysis examined the direct and indirect links between childhood abuse and clinical features with affective instability and impulsivity as mediators.

**Results:**

Affective instability significantly mediated the association between childhood abuse and earlier age of onset [effect estimate (*θ*)/standard error (SE): 2.49], number of depressive (*θ*/SE: 2.08) and manic episodes/illness year (*θ*/SE: 1.32), anxiety disorders (*θ*/SE: 1.98) and rapid cycling (*θ*/SE: 2.25). Impulsivity significantly mediated the association between childhood abuse and manic episodes/illness year (*θ*/SE: 1.79), anxiety disorders (*θ*/SE: 1.59), rapid cycling (*θ*/SE: 1.809), suicidal behaviour (*θ*/SE: 2.12) and substance misuse (*θ*/SE: 3.09). Measures of path analysis fit indicated an excellent fit to the data.

**Conclusions:**

Affective instability and impulsivity are likely part of the mechanism of why childhood abuse increases risk of poorer clinical course in bipolar disorder, with each showing some selectivity in pathways. They are potential novel targets for intervention to improve outcome in bipolar disorder.

## Introduction

Bipolar disorder is a multi-component illness (Rowland and Marwaha, 2018), and globally amongst the top 10 causes of disability (Murray and Lopez, 2007; Gore *et al*., 2011) with annual projected costs of £8 billion by 2020 (Knapp *et al*., 2011). The underlying risks and developmental pathways are poorly understood and new targets for treatment are needed. As in other psychiatric disorders, childhood abuse has been found to be a risk factor for the poorer course in bipolar disorder. In a recent meta-analysis, childhood maltreatment was shown to increase the odds of greater depression and mania severity and episode number, PTSD, anxiety disorders and earlier age of onset (Agnew-Blais and Danese, 2016). Childhood maltreatment in bipolar disorder is known to impact on brain function with changes in hippocampal and amygdala volumes (Janiri *et al*., 2017) and white matter integrity (Stevelink *et al*., 2018); it is also linked to changes in functional connectivity (Souza-Queiroz *et al*., 2016). The mechanisms underlying how childhood abuse can lead to poor illness course remain to be elucidated.

We and others have investigated whether affective instability could be an important aspect of this mechanism. Affective instability is linked to changes in the amygdala and salience networks (Broome *et al*., 2015*a*) and can be defined as rapid oscillations of intense affect with difficulties in regulating these or their behavioural consequences (Marwaha *et al*., 2013*a*, 2013*b*). Whilst affective instability is transdiagnostic (Broome *et al*., 2015*b*; Marwaha *et al*., 2018), it may be particularly important in bipolar disorder (Harrison *et al*., 2017). It is linked to the poor functional outcome (Strejilevich *et al*., 2013; Stange *et al*., 2016) and worse clinical course (Etain *et al*., 2013) in this condition. In our previous study, we found childhood trauma was significantly associated with higher affective instability levels in bipolar I, but not bipolar II disorder or major depression (Marwaha *et al*., 2016), demonstrating a plausible pathway. Aas *et al*. (2016) investigated this pathway and report that the link between childhood trauma and suicide attempts, anxiety disorders and mixed episodes in bipolar spectrum disorders (*n* = 342) is explained entirely by an indirect pathway via affective instability.

Impulsivity is correlated with, but distinct to affective instability and may also be a candidate pathology that might explain how childhood abuse exacts a consequence in bipolar disorder. It has been defined as a predisposition towards unplanned reactions, without regard to the negative consequences (Moeller *et al*., 2001). Childhood traumatic experiences are linked to impulsivity as well as suicidal thinking (Brodsky *et al*., 2001; Jiménez-Treviño *et al*., 2017). In turn, impulsivity is increasingly recognised as being strongly associated with bipolar disorder (Muhtadie *et al*., 2014; Dervic *et al*., 2015) as a state, as well as trait-like phenomena. It has been associated with abnormalities in orbital frontal white matter in bipolar disorder (Mahon *et al*., 2012), transition to first-episode bipolar disorder in youths with high reward-sensitivity (Ng *et al*., 2016), manic symptom severity (Najt *et al*., 2007) and poorer functioning (Lombardo *et al*., 2012). Whilst limited, the evidence indicates impulsivity is associated with earlier onset, more frequent episodes and a history of suicidal attempts (Swann *et al*., 2009) in bipolar disorder.

Thus, in summary, there is initial suggestive evidence that both affective instability and impulsivity may be important in explaining how childhood abuse impacts on clinical domains of bipolar disorder. Further evaluation of this hypothesis requires pathway modelling in a suitably large sample. We therefore aimed to examine whether childhood abuse was associated with clinical features in a large sample of people with bipolar I disorder, and whether any associations are mediated via measures of affective instability and/or impulsivity.

## Methods

The study was part of an on-going programme of research into the genetic and non-genetic determinants of bipolar disorder and related mood disorders (UK Bipolar Disorder Research Network, BDRN; https://www.bdrn.org) which has UK National Health Service Research Ethics Committee approval and local Research and Development approval in all participating areas. Full details of the study design are provided in our baseline paper (Marwaha *et al*., 2016)

### Subjects

Recruitment throughout the UK was via systematic and non-systematic methods. Systematic recruitment involved screening for potential participants through Community Mental Health Teams and Lithium clinics in the UK NHS. Clinical teams identified patients who met the research inclusion criteria and contacted them to invite them to participate in the research. Non-systematic recruitment involved advertising for volunteers, mainly on the BDRN website and through the UK-wide patient support organisation, Bipolar UK (https://www.bipolaruk.org/), which advertised the research via its newsletters, conferences and website.

Inclusion criteria were: aged at least 18 years; able to provide written informed consent; met DSM-IV criteria for bipolar I disorder; and mood symptoms onset before the age of 65 years. Individuals were excluded if they experienced affective illness only as a result of substance use or medical illness or were biologically related to another study participant. Participants included in the analyses reported here had completed measures of affective instability and impulsivity.

### Assessments

Participants were interviewed face-to-face using the Schedules for Clinical Assessment in Neuropsychiatry (SCAN) (Wing *et al*., 1990). Psychiatric and/or general practice (primary care) case-notes were also reviewed using a structured proforma. Data were recorded under headings representing premorbid background, onset of illness, each illness episode (including age, length of episode, symptom checklist, admission, section, level of functioning) and function and symptoms between episodes (often recorded in outpatient appointment summaries). Based on these, best-estimate lifetime diagnoses were made according to DSM-IV criteria, and the following eight lifetime clinical variables were rated for each participant:

(i). Suicidal behaviour, defined as the presence/absence of at least one suicide attempt;
(ii). Substance misuse, defined as at least harmful use of alcohol or illicit drugs. The negative consequences considered were continued use despite social, occupational, psychological or physical problems;
(iii). Rapid cycling, defined as the presence/absence of at least four affective episodes in any 12-month period;
(iv). Psychotic symptoms, defined as the presence/absence of one or more hallucination or delusion;
(v). Anxiety disorder, defined as the presence/absence of doctor diagnosis of any anxiety disorder (e.g. generalised anxiety disorder) recorded in medical case-notes or reported at interview, or significantly impairing anxiety episodes ascertained during the SCAN interview;
(vi). Age of onset, defined as the age of first impairment due to affective illness;
(vii). Mean number of episodes of depression per year over the lifetime of the illness;
(viii). Mean number of episodes of mania per year over the lifetime of the illness.

The variables were all rated using a combination of data obtained using the SCAN interview and data obtained from case-notes. They were chosen as a core set of lifetime clinical variables rated for the majority of participants and are commonly used clinical course variables in bipolar disorder research and a broader set than has been included in previous studies.

In cases of doubt, diagnosis and clinical ratings were made blindly by at least two research team members and consensus was reached via discussion where necessary. Inter-rater reliability was assessed using 20 random cases. Mean *κ* statistics were 0.85 for DSM-IV diagnoses and ranged between 0.81 and 0.99 for categorical clinical variables. Mean intra-class correlation coefficients were between 0.91 and 0.97 for continuous clinical variables. Staff involved in assessments were all research psychologists or psychiatrists.

Information about adverse childhood life events (ACLEs) was gathered using a bespoke instrument, Childhood Life Events Questionnaire (CLEQ), developed by the BDRN (see Upthegrove *et al*., 2015 for further information). The CLEQ was administered verbally to all participants following the SCAN interview once there had been the opportunity for rapport to be established. Participants were asked if they experienced one or more of a list of 12 childhood events, not including abuse, before the age of 16 years. We chose to not specifically ask about experiences of childhood abuse due to the sensitive nature of such events. Instead, participants were given the opportunity to disclose additional events by being asked ‘Are there any other significant life events you experienced as a child that are not mentioned above?’ Case notes were also reviewed for any mention of ACLEs including abuse. Participants also completed the Brief Life Events Questionnaire (BLEQ) asking about severe life events based on the list proposed by Brugha *et al*. (1985). An open question was added to the questionnaire asking participants ‘Do you think that there is anything that has happened to you during your life which has contributed to you becoming unwell?’ This was also examined for evidence of ACLEs including abuse. These sources of information were combined to code the presence or absence of any abuse (sexual and/or physical and/or emotional) categorically.

Affective instability was measured using the Affective Lability Scale-Short Form (ALS-SF) (Oliver and Simons, 2004), which has been used in multiple relevant previous studies (Henry *et al*., 2001; Henry *et al*., 2008; Aas *et al*., 2015). It is an 18-item self-report questionnaire measuring rapid changes from euthymic mood to other emotional states including elation, depression and anger. Total score ranges from 18 to 72, with higher scores indicating increased affective instability.

The self-report Barratt Impulsiveness Scale version-11 (BIS) (Patton *et al*., 1995) was used to assess impulsivity. It has 30 items, with total scores ranging from 30 to 120, with higher scores indicating increased impulsivity.

The ALS-SF and BIS were sent to previously recruited BDRN participants as part of a questionnaire mailshot with a reminder a month later.

In order to account for the current mood state in measures of affective instability and impulsivity, participants also completed measures of current depression and mania symptoms at the same time as measures of affective instability and impulsivity. The measures completed were the Beck Depression Inventory (BDI) (Beck and Steer, 1987) and Altman Self-Rating Mania Scale (AMS) (Altman *et al*., 1997).

### Analysis

The data were initially analysed using *SPSS* (version 24.0). A subsequent path analysis was carried out with *Mplus* software (version 8.1). We followed a step-by-step approach to develop our model of how childhood abuse might impact on clinical domains of bipolar disorder via affective instability, or impulsivity, or both. First, associations between childhood abuse, ALS-SF score, BIS score and each of the clinical variables were analysed univariately using either linear or binary logistic regression. Associations between possible confounders (age, sex, education level, method of recruitment) and the clinical variables were also analysed using linear or binary logistic regression. Secondly, the presence of childhood abuse, ALS-SF score and BIS score were entered together as explanatory variables to investigate associations with each of the clinical variables, whilst controlling for demographic confounders. Employment status was closely associated with educational attainment in the sample, and therefore we adjusted for education only in order to reduce the risks of multicollinearity. Thirdly, current mood state was adjusted for.

For the path analysis, childhood abuse (presence/absence), ALS-SF score and BIS score were entered as explanatory variables, and childhood abuse was allowed to act on the clinical outcomes either directly or via ALS-SF score or BIS score. Seven clinical outcomes were included. Paths from current mood state to ALS-SF and BIS score were included to account for the effects of current mood state on their ratings. Paths from the demographic confounders to the clinical variables were included, and the covariance between ALS-SF score and BIS score was explicitly modelled, as previous research suggests they are correlated (Peters *et al*., 2016). As in previous path analyses that incorporated categorical clinical outcome variables (Etain *et al*., 2017*b*; Marwaha *et al*., 2017), the WLSMV (weighted least squares mean and variance adjusted) estimator was used, which produces probit coefficients. Confidence intervals were estimated by computing 1000 bootstrap samples. After estimation, non-significant (*p* > 0.05) paths were dropped from the model and the model was re-estimated.

## Results

### Sample description

The sample comprised 923 individuals with bipolar I disorder: 74.9% (*n* = 692) were female; 44.9% (*n* = 415) had completed higher education; mean age at interview was 49 years (s.d. = 11.5); 21.8% (*n* = 201) was recruited systematically; 16.3% (*n* = 150) had a history of childhood abuse [10.1% (*n* = 93): sexual abuse, 8.8% (*n* = 81): physical abuse, 3.1% (*n* = 29): emotional abuse]. In total, 4.9% of cases (*n* = 45) reported experiencing more than one type of childhood abuse (30% of those reporting any abuse).

The response rate for completion of the ALS and BIS was 37%. There were significant sociodemographic differences between the responders and non-responders with an over-representation among the responders of female sex (*p* < 0.001) and having higher education (*p* < 0.001), and responders being older (*p* < 0.001). However, there were no significant differences between responders and non-responders in mean age at onset (24 years in both), frequency of suicidal behaviour, substance misuse, rapid cycling, psychosis or mean DSM-IV Global Assessment of Functioning scores in the worst episode of mania and depression in each group.

The mean score (standard deviation) on the BDI and AMS was 13.4 (10.9) and 3.3 (3.4), respectively. Further descriptive data on these measures can be found in our baseline paper (Marwaha *et al*., 2016). Mean ALS-SF and BIS scores were 38.9 (s.d. = 13.5) and 66.0 (s.d. = 11.9), respectively. History of suicidal behaviour was present in 48.8% of cases (*n* = 450), substance misuse in 38.1% (*n* = 352), rapid cycling in 20.4% (*n* = 188), psychotic symptoms in 68.7% (*n* = 634) and anxiety disorder in 53.2% (*n* = 491). Mean age of illness onset was 23.6 years (s.d. = 9.2), mean number of episodes of depression per illness year was 0.54 (s.d. = 0.67) and mean number of episodes of mania per illness year was 0.44 (s.d. = 0.54).

### Associations between childhood abuse, affective instability and impulsivity, and clinical features

Table 1 shows the association [odds ratios (ORs) or *β* coefficients] between childhood abuse and each of the clinical variables, prior to correction for potential confounding variables. In particular, childhood abuse was significantly associated with the presence of suicidal behaviour, substance misuse, rapid cycling and anxiety disorder, as well as earlier age of illness onset (by, on average, 4.2 years), and increased mean number of episodes of depression and mania per illness year.

**Table 1.** Relationships between childhood abuse, ALS-SF score, BIS score, potential confounding variables and clinical variables

	Suicidal behaviour OR (95% CI)	Substance misuse (OR, 95% CI)	Rapid cycling (OR, 95% CI)	Psychotic symptoms (OR, 95% CI)	Anxiety disorder (OR, 95% CI)	Age of illness onset (*β*, 95% CI)	# eps depression/year (*β*, 95% CI)	# eps mania/year (*β*, 95% CI)
Childhood abuse	2.584**** (1.763–3.789)	2.478**** (1.737–3.536)	1.751* (1.124–2.728)	0.923 (0.587–1.452)	2.618**** (1.785–3.842)	−4.196*** (−5.830 to −2.562)	0.293*** (0.172–0.415)	0.255*** (0.157–0.353)
ALS-SF score (increment)	1.030**** (1.019–1.040)	1.027**** (1.017–1.038)	1.058**** (1.042–1.073)	0.996 (0.984–1.008)	1.032**** (1.022–1.042)	−0.168*** (−0.211 to −0.124)	0.012*** (0.009–0.015)	0.008*** (0.006–0.011)
BIS score (increment)	1.038**** (1.026–1.050)	1.039**** (1.027–1.051)	1.062**** (1.045–1.079)	0.989 (0.975–1.003)	1.036**** (1.024–1.048)	−0.164*** (−0.214 to −0.114)	0.012*** (0.008–0.016)	0.011*** (0.008–0.014)
Female sex	1.369* (1.008–1.860)	0.750 (0.554–1.016)	1.130 (0.762–1.674)	1.216 (0.835–1.772)	1.479** (1.097–1.994)	−1.532* (−2.933 to −0.131)	0.115* ( 0.009–0.221)	−0.015 (−0.099 to 0.069)
Completed higher education	0.658*** (0.503–0.862)	1.143 (0.872–1.498)	0.784 (0.555–1.107)	0.960 (0.683–1.347)	0.936 (0.719–1.219)	0.315 (−0.926 to 1.556)	−0.044 (−0.136 to 0.049)	0.006 (−0.066 to 0.078)
Systematically recruited	1.131 (0.820–1.559)	0.933 (0.675–1.290)	0.871 (0.571–1.327)	0.596 (0.408–0.870)	0.685* (0.500–0.938)	0.605 (−0.881 to 2.091)	−0.037 (−0.150 to 0.076)	−0.055 (−0.144 to 0.035)
Age at interview (increment)	0.992 (0.981–1.003)	0.963**** (0.951–0.974)	0.983* (0.968–0.998)	0.979*** (0.964–0.994)	0.994 (0.983–1.005)	0.256**** (0.206–0.306)	−0.013**** (−0.017 to −0.009)	−0.011**** (−0.014 to −0.008)
BDI score (increment)	1.042**** (1.029–1.056)	1.043**** (1.030–1.056)	1.064**** (1.046–1.083)	0.994 (0.979–1.010)	1.038**** (1.024–1.051)	−0.154**** (−0.210 to −0.098)	0.015**** (0.011–0.019)	0.010**** (0.007–0.014)
AMS score (increment)	1.051* (1.011–1.092)	1.040* (1.001–1.081)	1.091*** (1.039–1.145)	0.997 (0.949–1.048)	1.054** (1.013–1.096)	−0.385**** (−0.563 to −0.207)	0.017* (0.004–0.031)	0.016**** (0.006–0.027)

OR, odds ratio; CI, confidence interval; ALS-SF, Affective Lability Scale-Short Form; BIS, Barrett Impulsiveness Scale; # eps depression/year, mean number of episodes of depression per illness year; # eps mania/year, mean number of episodes of mania per illness year. For ALS-SF, BIS, Age at interview, BDI and AMS, ORs and *β* coefficients are those associated with a one-point increment in score/age. A statistically significant result are shown in bold.

*p < 0.05; **p < 0.01; ***p < 0.005; ****p < 0.001.

Increased affective instability and impulsivity were significantly associated with the presence of suicidal behaviour, substance misuse, rapid cycling, anxiety disorder and increased mean number of episodes of depression and mania per illness year, as well as with earlier age of illness onset.

Female sex was significantly associated with suicidal behaviour, anxiety disorder, younger age of illness onset and increased number of depressive episodes per illness year. Higher education was significantly associated with the absence of suicidal behaviour. Age at interview and current elevated and depressive mood state were significantly associated with most clinical features. Childhood abuse was not associated with psychotic symptoms.

### Associations after adjustment for demographic variables and current mood state

Table 2 shows the association between the presence of childhood abuse, affective instability and impulsivity, now entered together as explanatory variables, and clinical features, controlling for sex, education, recruitment type and age at interview. Affective instability was no longer significantly associated with suicidal behaviour or substance misuse, whilst impulsivity was no longer significantly associated with earlier age of illness onset or number of episodes of depression per year. Childhood abuse was no longer significantly associated with the presence of rapid cycling.

**Table 2.** Relationships between childhood abuse, ALS-SF score and BIS score (entered together) and the clinical variables, controlling for demographic variables^a^

Predictors entered in same model	Suicidal behaviour (OR, 95% CI)	Substance misuse (OR, 95% CI)	Rapid cycling (OR, 95% CI)	Psychotic symptoms (OR, 95% CI)	Anxiety disorder (OR, 95% CI)	Age of illness onset (*β*, 95% CI)	# eps depression/ year (*β*, 95% CI)	# eps mania/ year (*β*, 95% CI)
Childhood abuse	2.072**** (1.368–3.141)	2.133**** (1.432–3.176)	1.181 (0.710–1.964)	0.770 (0.470–1.262)	1.976**** (1.303–2.996)	−1.965* (−3.585 to −0.346)	0.160* (0.034–0.286)	0.140** (0.040–0.239)
ALS-SF score (increment)	1.013 (1.000–1.026)	1.010 (0.997–1.024)	1.043**** (1.024–1.061)	1.003 (0.987–1.019)	1.021**** (1.008–1.034)	−0.124**** (−0.177 to −0.072)	0.008**** (0.004–0.012)	0.004* (0.000–0.007)
BIS score (increment)	1.024*** (1.008–1.039)	1.029**** (1.013–1.044)	1.035**** (1.015–1.055)	0.985 (0.967–1.003)	1.021*** (1.006–1.036)	−0.041 (−0.102 to 0.019)	0.003 (−0.001 to 0.008)	0.006*** (0.002–0.010)

OR, odds ratio; CI, confidence interval; ALS-SF, Affective Lability Scale-Short Form; BIS, Barrett Impulsiveness Scale; # eps depression/year, mean number of episodes of depression per illness year; # eps mania/year, mean number of episodes of mania per illness year.

a Sex, education level, age at interview, recruitment method.

*p < 0.05; **p < 0.01; ***p < 0.005; ****p < 0.001

Table 3 shows the associations after additionally controlling for current mood state (BDI and AMS scores), which were somewhat weakened, but the pattern of significance remained the same, except in two cases: affective instability was no longer significantly associated with the presence of an anxiety disorder or the number of episodes of mania per year of illness.

**Table 3.** Relationships between childhood abuse, ALS-SF score and BIS score (entered together) and the clinical variables, controlling for demographic variables^a^, and current mood state^b^

Predictors entered in same model	Suicidal behaviour (OR, 95% CI)	Substance misuse (OR, 95% CI)	Rapid cycling (OR, 95% CI)	Psychotic symptoms (OR, 95% CI)	Anxiety disorder (OR, 95% CI)	Age of illness onset (*β*, 95% CI)	# eps depression/ year (*β*, 95% CI)	# eps mania/ year (*β*, 95% CI)
Childhood abuse	2.067**** (1.347–3.172)	1.991**** (1.323–2.998)	1.029 (0.605–1.751)	0.786 (0.470–1.314)	1.845*** (1.205–2.826)	−1.884* (−3.547 to −0.222)	0.142* (0.012–0.271)	0.124* (0.022–0.227)
ALS-SF score (increment)	1.005 (0.991–1.020)	1.000 (0.985–1.015)	1.028** (1.008–1.049)	1.001 (0.983–1.019)	1.014 (1.000–1.029)	−0.099**** (−0.158 to −0.040)	0.005* (0.001–0.010)	0.002 (−0.001 to 0.006)
BIS score (increment)	1.018* (1.002–1.034)	1.021** (1.005–1.038)	1.023* (1.002–1.044)	0.988 (0.969–1.008)	1.017* (1.001–1.033)	−0.025 (−0.089 to 0.039)	0.001 (−0.004 to 0.007)	0.005** (0.001–0.009)

OR, odds ratio; CI, confidence interval; ALS-SF, Affective Lability Scale-Short Form; BIS, Barrett Impulsiveness Scale; # eps depression/year, mean number of episodes of depression per illness year; # eps mania/year, mean number of episodes of mania per illness year.

a Sex, education level, age at interview, recruitment method.

b AMS (Altman Mania Scale), BDI (Beck Depression Inventory).

*p < 0.05; **p < 0.01; ***p < 0.005; ****p < 0.001.

### Direct and indirect paths from childhood abuse to clinical outcomes

**Fig. 1.**
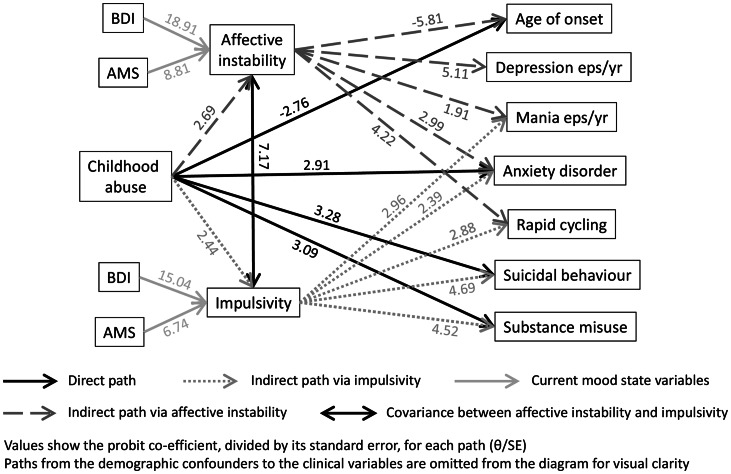
Pathways between childhood abuse and clinical outcomes in bipolar disorder

The final path analysis model is shown in Fig. 1. Psychotic symptoms were excluded in the path analysis given the lack of significant associations in the preceding analyses. The model incorporated direct paths from childhood abuse to each of the clinical outcomes, and indirect paths via affective instability or impulsivity. To facilitate comparisons between effect sizes, we report effect estimates divided by their standard errors.

Measures of path analysis fit indicated excellent fit to the data: RMSEA (root mean square error of approximation) = 0.041 (90% CI 0.030–0.051); SRMR (standardised root mean square residual) = 0.077; TLI (Tucker Lewis index) = 0.943; and CFI (comparative fit index) = 0.974. Typically, RMSEA and SRMR less than 0.08, and TLI and CFI above 0.90 are taken as indicating a good model fit (Hu and Bentler, 1999; Hooper *et al*., 2008). The covariance between BIS score and ALS-SF score was retained, as were the paths from the current mood state variables to BIS and ALS-SF score.

For suicidal behaviour, the direct path from childhood abuse was retained (*θ*/SE: 3.284), as was an indirect path via impulsivity (*θ*/SE: 2.123). For substance misuse, a direct path (*θ*/SE: 3.089) and an indirect path (*θ*/SE: 2.095) via impulsivity were also retained. For rapid cycling, the direct path from childhood abuse was dropped, but the indirect paths via affective instability (*θ*/SE: 2.252) and impulsivity (*θ*/SE: 1.809) were retained. For the presence of an anxiety disorder, the direct path (*θ*/SE: 2.913) and both indirect paths (affective instability: *θ*/SE: 1.979; impulsivity: *θ*/SE: 1.587) were retained.

For age of illness onset, the direct path from childhood abuse was retained (*θ*/SE: −2.758), as was the indirect path via affective instability (*θ*/SE: −2.488) with abuse being linked to an earlier age of onset. For the number of episodes of depression per year, only the indirect path via affective instability was retained (*θ*/SE: 2.077). Finally, for the number of episodes of mania per year, the direct path was dropped, but the indirect paths via affective instability (*θ*/SE: 1.319) and impulsivity (*θ*/SE: 1.792) were retained.

## Discussion

### Main findings

We examined the inter-connections between childhood abuse, affective instability, impulsivity and multiple clinical domains, indicative of greater illness severity, in 923 people with bipolar I disorder. Furthermore, we identified potential mechanistic pathways between childhood abuse and these clinical domains. To our knowledge, this is the largest study to date examining these questions. There are a number of important and robust findings.

Childhood abuse more than doubled the odds of suicidal behaviour, substance misuse and having an anxiety disorder and was strongly associated with an earlier age of illness onset, as well as increased number of episodes of depression or mania. A meta-analysis of previous studies indicates a connection between childhood abuse and clinical course in bipolar disorder, but many previous investigations have not controlled for important confounders (Daruy-Filho *et al*., 2011). Our results were robust to adjustment for socio-demographic variables and current mood state. Clearly, childhood abuse is extremely damaging in the life course of people with bipolar disorder, and needs to be the focus of much greater attention than is currently the case in this condition.

Affective instability as well as impulsivity, both of which can be thought of as trait-like factors manifesting during childhood and adolescent development, were independently associated with rapid cycling, presence of an anxiety disorder and number of episodes of mania per illness year. Only affective instability explained earlier age of illness onset and mean number of episodes of depression per illness year, whilst impulsivity was linked to suicidal behaviour and substance misuse in the regression modelling. Using a large sample size, our findings therefore support the developing neuroscience literature (Mahon *et al*., 2012; Trost *et al*., 2014; Broome *et al*., 2015*a*), that dimensions of affect and response control are important in bipolar disorder.

Our path analysis demonstrates affective instability and impulsivity form part of the pathway from childhood abuse to multiple clinical domains in bipolar disorder. The pattern of associations suggests some sensitivity in the pathways in which affective instability and impulsivity are individually important. Thus, affective instability selectively explained the path between childhood abuse and more lifetime depressive episodes and earlier age of illness onset (in the former, in the absence of any direct association). We have previously shown that affective instability is common in depression (Marwaha, 2013b; Balbuena *et al*., 2016) and prospectively links to new onset cases (Marwaha *et al*., 2015) and this current study adds weight to evidence of this trait's importance in major mood disorders. It appears that affective instability acts to bring forward illness onset in a group that have already been made vulnerable because of childhood abuse, with affect change becoming more labile, so that episodes of mania or depression develop. Affect or mood instability is common in bipolar disorder outside of mania and depression episodes (Henry *et al*., 2008; Moore *et al*., 2014), and is part of the putative pathway to first-episode bipolar illness (Howes *et al*., 2011; Berk *et al*., 2017).

Higher impulsivity selectively mediated the path between childhood abuse and suicidal behaviour, and substance misuse, as well as there being a direct link. Whilst abuse and impulsivity have been previously linked individually to suicidal behaviour in a number of studies (Brodsky *et al*., 2001; Swann *et al*., 2005; Liu *et al*., 2017), though not all (Ugur *et al*., 2014), we demonstrate how they might be connected in a putative mechanism. Our findings are also supported by neurobiological findings connecting impulsivity and suicidal behaviour (Mann *et al*., 2001; Matsuo *et al*., 2010).

As well as showing some mechanistic selectivity in pathways, affective instability and impulsivity both appeared to be important in trajectory to other clinical parameters. The path from childhood abuse to anxiety disorders, lifetime number of mania episodes as well as to rapid cycling was mediated by both impulsivity and affective instability. In the case of rapid cycling, the association was largely indirect indicating that to some degree rapid cycling is contingent on the development of both affective instability and impulsivity in people who have suffered childhood abuse.

Given that mood symptoms mediate traumatic events and the new onset of psychotic symptoms in the general population (Catone *et al*., 2015), it was interesting that our path analysis demonstrated no connection between abuse and psychotic symptoms in people with bipolar disorder. There is a prevalent view that psychotic symptoms in bipolar disorder indicate a more severe form of the illness but this has been challenged by a recent study (Burton *et al*., 2018) and they were recently found not to be important in the association between childhood abuse, cannabis use and hypomania (Marwaha *et al*., 2017).

### Strengths and limitations

This study had several strengths. It is the largest study to date investigating childhood abuse, affective and impulsive traits and clinical parameters in bipolar I disorder. We focussed on bipolar I only, building on our previous study results. We examined both affective instability and impulsivity in our modelling to achieve a complex understanding of the mechanism. Furthermore, we controlled for current mood state in our analyses reducing the possibility that either affective instability or impulsivity were simply manifestations of mental state, given that these measures were completed at the same time.

Our results appear to partly substantiate the only other previous study in this area. Etain *et al*. (2017*b*) report that in a sample of 485 people with bipolar disorder, emotional abuse appeared to be most critical, and that affective intensity and attitudinal hostility mediated its connection to suicide attempts, whilst impulsivity mediated its association with substance misuse. Whilst our approach is similar to this previous work, this study adds significantly to the field. There are very few path analyses available in bipolar disorder samples, and studies of the impact of trauma, both in childhood and in adults in bipolar disorder, are surprisingly limited in comparison to research in other disorders. Our sample also enabled the inclusion of a greater number of studied clinical variables than the work of Etain *et al*. (2017*a*, 2017*b*), in part, because of the larger sample size. Finally, our work also adds value in that we can be confident that our results were not substantially confounded by the impact of current mood state on ratings of affective instability or impulsivity.

The prevalence of childhood abuse in the current study was somewhat lower than in other samples that have used the childhood trauma questionnaire (CTQ) (Romero *et al*., 2009; Li *et al*., 2014), though interestingly broadly similar to a study sampling UK patients that used the CTQ (Watson *et al*., 2014). Information about childhood trauma was comprehensive with data collection triangulated from interview self-report and using information from medical case-notes. We do not know whether the relatively low rates in this study represent an issue of sensitivity. Some previous work suggests that the impact of emotional abuse on clinical outcomes may be particularly important (Etain *et al*., 2017*b*). However, we did not find a statistically significant association between emotional abuse in bipolar disorder and affective instability in our previous analysis (Marwaha *et al*., 2016) using the same dataset as in the current analysis. Therefore, we chose to examine ‘any abuse’ as opposed to the impact of different types of abuse.

There are also several limitations. The analyses are not based on prospective data and therefore we cannot be sure that path associations are causal, though the results have biological and psychological plausibility, and build on a significant background literature. In addition, given that childhood abuse was demarcated as occurring before age 16 years indicates that the exposure occurred years before the outcomes of interest. Traits such as affective instability and impulsivity are thought to develop and stabilise in adolescence (Etain *et al*., 2008) and therefore there is some validity in our assertion that they are likely putative mediators. However, it remains possible that childhood traumatic experiences may be a consequence of behavioural or emotional functioning linked to a predisposition to bipolar disorder (Etain *et al*., 2008). Also, in this study, retrospective assessment bias may have played a part in our results.

The response rate to the completion of ALS-SF and BIS was 37%. There were no significant clinical differences between responders and non-responders and thus it is unlikely there is bias related to illness severity of bipolar disorder in responders. The sociodemographic differences between the groups are likely to represent the bias inherent in questionnaire research; that is females, people with higher education and older people more likely to respond. However, our analyses are based on a large well-characterised sample of people with bipolar disorder living in the community.

We controlled for sex, education, recruitment type and age at interview in the statistical analyses. We did not adjust for family psychiatric history or medication as these data were not available for the majority of participants. As in all mental health research studies that rely on longitudinal (retrospective) clinical assessment, recall bias may have been a factor in measurement.

## Conclusions

Despite these limitations, our study supports the hypothesis that affective instability and impulsivity are important in bipolar I disorder and form part of the putative pathway that explains why individuals who have suffered from childhood abuse have a greater severity of illness. Each mediator shows some discrimination in the pathways in which it is important. We need better strategies for the prevention of childhood abuse. Our results also point to the potential of affective instability and impulsivity as new novel targets for intervention. The challenge now is to develop and test these in order to reduce the morbidity caused by bipolar disorder.
